# Therapeutic Effects of Olive and Its Derivatives on Osteoarthritis: From Bench to Bedside

**DOI:** 10.3390/nu9101060

**Published:** 2017-09-26

**Authors:** Kok-Yong Chin, Kok-Lun Pang

**Affiliations:** 1 Department of Pharmacology, Universiti Kebangsaan Malaysia Medical Centre, Cheras 56000, Malaysia; 2 Biomedical Science Programme, School of Diagnostic and Applied Health Sciences, Faculty of Health Sciences, Universiti Kebangsaan Malaysia, Kuala Lumpur 50300, Malaysia; pangkoklun@gmail.com

**Keywords:** autophagy, cartilage, chondrocyte, hydroxytyrosol, inflammation, joint, oleocanthal, oleuropein, sirtuin-1, tyrosol

## Abstract

Osteoarthritis is a major cause of morbidity among the elderly worldwide. It is a disease characterized by localized inflammation of the joint and destruction of cartilage, leading to loss of function. Impaired chondrocyte repair mechanisms, due to inflammation, oxidative stress and autophagy, play important roles in the pathogenesis of osteoarthritis. Olive and its derivatives, which possess anti-inflammatory, antioxidant and autophagy-enhancing activities, are suitable candidates for therapeutic interventions for osteoarthritis. This review aimed to summarize the current evidence on the effects of olive and its derivatives, on osteoarthritis and chondrocytes. The literature on animal and human studies has demonstrated a beneficial effect of olive and its derivatives on the progression of osteoarthritis. In vitro studies have suggested that the augmentation of autophagy (though sirtuin-1) and suppression of inflammation by olive polyphenols could contribute to the chondroprotective effects of olive polyphenols. More research and well-planned clinical trials are required to justify the use of olive-based treatment in osteoarthritis.

## 1. Introduction

Osteoarthritis (OA) is a common degenerative disease of the joints among the elderly. It is induced by accumulated micro- and macro-injuries that lead to a maladaptive repair response of the joints [1]. Current perspective holds that molecular derangements, characterised by abnormal joint metabolism, precede anatomical/physiological derangements of the joint, which are marked by cartilage degradation, increased subchondral bone remodelling, osteophyte formation and joint inflammation [1,2]. This ultimately translates to clinical manifestations of OA, which are marked by joint pain, tenderness, stiffness, impaired movement, crepitus and effusion [1,2]. The worldwide prevalence of knee OA is 3.8% (95% uncertainty interval (UI) 3.6% to 4.1%) and the prevalence of hip OA is 0.85% (95% UI 0.74% to 1.02%). Despite not being a fatal disease, it constituted the 11th largest cause of global disability (the 15th in 1990) and the 38th largest contributor of disability-adjusted life years (the 49th in 1990), in 2010 [3]. The annual mean direct and indirect medical costs of OA per patient were estimated to be €2013 (range €0.7–12), but this estimate was skewed towards Western countries [4]. 

Aging is the major risk factor for OA [5,6]. Under normal conditions, chondrocytes are responsible for the synthesis, regeneration and maintenance of the cartilage matrix. However, senescence of chondrocytes (chondrosenescence) occurs as a consequence of aging. This process compromises the ability of the chondrocytes to maintain and repair the articular cartilage tissue [5,6]. Chondrosenescence, with the prevailing harmful biomechanical stress, leads to irreversible chondrocyte cell death, which ultimately leads to cartilage damage, matrix depletion and loss of cartilage cellularity [7,8,9,10].

Several pathways are involved in the pathogenesis of OA. Numerous studies have reported the involvement of inflammation in the progression of OA. Mechanical damage can cause a localized inflammatory response of the joint, marked by increased pro-inflammatory mediators, such as interleukin-1β (IL-1β), interleukin-6 (IL-6), tumour necrosis factor-α (TNF-α), nitrite oxide (NO) and prostaglandin E_2_ (PGE_2_) in the joint space [11,12,13,14,15]. This inflammatory response further exaggerates cartilage tissue damage via oxidative stress and damage, thus forming a vicious self-destructive cycle. Oxidative stress is also related to OA, as evidenced by an upregulation of inducible NO synthase (iNOS) and nicotinamide adenine dinucleotide phosphate oxidase in chondrocytes [6]. These enzymes produce high levels of reactive oxygen and nitrogen species (ROS and RNS), including NO, superoxide anion, peroxynitrite and hydrogen peroxide (H_2_O_2_) [16,17,18,19,20,21]. The cellular antioxidant enzymes have been found to be compromised in animal models and patients with OA [20,22,23,24,25,26]. An imbalance between oxidants and antioxidants results in oxidative damage, endoplasmic reticulum stress and mitochondrial dysfunction in chondrocytes (intrinsic pathway of apoptosis) [8,27,28,29], which subsequently leads to chondrocytic differentiation or apoptosis [30]. In addition the overexpression of death receptor 5 and TNF-related apoptosis-inducing ligand in the cartilage of OA patients [31], Fas-induced apoptosis of chondrocytes [6,32] may also contribute to the pathogenesis of OA. 

The cartilage layer is avascular and alymphatic, with limited blood and oxygen supplies [33,34,35]. Chondrocytes adapt to this hypoxic condition via the constitutively activated 5′ adenosine monophosphate-activated protein kinase/Sirtuin-1 (AMPK/SIRT-1) signalling pathway [34,36,37,38]. AMPK signalling is important for energy production and regulation [36]. Recent evidence showed that SIRT-1 activation protects chondrocytes from apoptosis and radiation-induced senescence by improving mitochondrial function [39,40,41]. Both AMPK and SIRT-1 have been reported to inhibit inflammation and cartilage catabolism [39,40,41,42]. Studies showed that resveratrol (a SIRT-1 activator) can protect chondrocytes from oxidative stress, inflammation and apoptosis [43,44,45,46]. Furthermore, chondrocytes are highly dependent on autophagy as a reparatory mechanism during cellular damage, due to their limited mitotic capacity [33,47]. Autophagy removes any damaged or dysfunctional organelles, without compromising the cartilage cellularity [36,48]. Previous studies have demonstrated that autophagy processes and AMPK/SIRT-1 activities were compromised, via experimental models and patients with OA [48,49,50]. Conversely, AMPK and SIRT-1 activation could improve the progression of OA via autophagy induction [36,51,52]. Rapamycin (an autophagy inducer) and glucosamine (a joint supplement) were demonstrated to improve clinical signs of OA in experimental animals, by promoting autophagy of chondrocytes [53,54]. Glucosamine sulphate also significantly improves pain and joint function in the knees of OA patients [55,56,57], but its effect against joint space narrowing is moderate [35,58,59]. 

Circulating markers of cartilage catabolism can be used to monitor the progression of OA [60,61,62,63]. These markers include enzymes that are involved in matrix degradation in response to chondrocyte apoptosis, such as collagenases (matrix metalloproteinase (MMP)-1, -3, -8, and -13), gelatinases (MMP-2 and MMP-9) and aggrecanase [22,64,65,66]. The proinflammatory cytokines, such as TNF-α and IL-1β, upregulate these MMPs through signal transduction pathways [37,67]. In addition, ROS also cleave collagen and aggrecan in the cartilage matrix directly, via oxidation, nitrosylation, nitration or chlorination [6,22,30,64]. As a result, collagen and aggrecan levels in synovial fluid increase. These cartilage degradation products can further promote cartilage inflammation, chondrocyte apoptosis and ROS production, via a positive feedback loop [6,8,9]. Other cartilage proteins, such as lubricin and β-defensin-4, are also potential biomarkers for OA. Lubricin, or proteoglycan 4, is a type of glycoprotein that serves as a boundary lubricant between cartilage surfaces, temporomandibular joint discs and tendons [9,68,69]. Lubricin expression is lower in senescent or OA chondrocytes [69]. The inflammatory process in OA also upregulates β-defensin-4, an antimicrobial peptide, which can break down cartilage matrix by increasing MMP activity [70]. Higher levels of β-defensin-4 were found in chondrocytes isolated from OA patients in either monolayer culture or scaffold [70,71]. These pathways involved in chondrocyte apoptosis can be targeted for novel OA treatments [6]. 

In this context, olive oil and its active components (including its biologically-active polyphenols—hydroxytyrosol, tyrosol, oleocanthal and oleuropein) serve as potential candidates for the treatment of OA. Olive oil and its derivatives have demonstrated promising antioxidant and anti-inflammatory properties in isolated erythrocytes, in vitro cultured cells [72,73,74,75,76,77,78], exercise-exhausted rat skeletal muscle [79] and other animal disease models [80,81,82,83,84]. Furthermore, hydroxytyrosol was previously shown to improve mitochondrial respiration and reduce oxidative stress in the brain of db/db mice via AMPK activation [85]. Tyrosol and oleuropein were reported to decrease oxidative damage in cultured cells [86,87] and in a rat model of myocardial ischemia, by upregulating the expression of SIRT-1 and its nuclear translocation [88]. A recent study also reported that daily extra-virgin olive oil (EVOO) intake for six months improved synaptic integrity, with lower insoluble protein aggregation, via autophagy activation, in a rat model of Alzheimer’s disease [89]. On the other hand, diets based on olive oil have reduced postprandial oxidative stress and inflammation in human studies [90,91,92]. These findings suggest that olive oil and its derivatives are potential therapeutic agents for inflammatory diseases like OA.

This review aimed to summarise the current evidence on the effects of olive oil and its derivatives on OA. A literature search was performed by the authors within the period of 1–31 July 2017 using the keywords ‘olive OR tyrosol OR hydroxytyrosol OR oleocanthal OR oleuropein’ AND ‘osteoarthritis OR cartilage OR chondrocyte’ in Pubmed and Scopus. Original research articles on human clinical trials, animals and cell culture studies, published in English, from the inception of the databases to the last date of the literature search (31 July 2017) were included. Studies on rheumatoid arthritis were excluded from this review. Fourteen studies were included for the final analysis. 

## 2. Effects of Olive and Its Derivatives in Animal Models of OA 

There are various methods to induce OA in experimental animals, such as injection of chemicals to induce collagen degradation or apoptosis of chondrocytes, surgical manoeuvres to destabilize the joint, the use of animals that have developed spontaneous OA and genetically modified animals. The animals of choice range from rodents to rabbits, dogs and monkeys. Each OA model and animal species has its pros and cons [93]. The studies included in this review used rodents and rabbits. OA developed spontaneously, or was induced using surgical methods. Progression of the disease was determined using histological assessments based on standard guidelines, collagen or glycoprotein expression in the cartilage layer, or serum biomarkers of cartilage degradation.

Gong et al. supplemented water extract of olive leaf in drinking water (500 mg/kg/day) in 16-week old New Zealand rabbits, through surgically-drilled holes at their stifle joints, for three weeks [94]. The surgery caused an invasion of macrophages and neutrophils in the cartilage layer of untreated rabbits, indicating an inflammatory process [94]. The subchondral bone also showed proliferation of osteoclasts, the bone-resorbing cells, indicating increased bone remodelling and subsequent bone damage [94]. Histological analyses showed a greater degree of healing in the supplemented rabbits relative to the untreated group. Proliferating chondrocytes, matured cartilage tissue and proteoglycans were present at the injured sites of supplemented rabbits [94]. The proliferation of osteoclasts at the subchondral bone was averted by using water extract of olive [94]. The researchers did not characterize the compounds found in the water extract used in this study, although the presence of hydroxytyrosol was suggested. The olive leaf extract was postulated to be anti-inflammatory, but pro-inflammatory cytokine levels were not examined. Nevertheless, this study provided clues that olive leaf extract possesses joint-protective properties. 

Extra-virgin olive oil (EVOO), commonly consumed in Mediterranean diets, contains a high amount of polyphenols (for example, oleuropein, tyrosol and hydroxytyrsosol) and unsaturated fatty acids (for example: oleic and linoleic fatty acid), because its production does not involve thermal and physical alternation of the oil [95,96]. Musumeci et al. compared the effects of mild physical activities (treadmill training), an EVOO-enriched diet, and the combination of both, in a rat model of OA, which was induced through anterior cruciate ligament transection for eight weeks [97]. The fat component of the experimental diet (30% of the total weight of the diet) was replaced with EVOO [97]. The combination of treadmill training and the EVOO-enriched diet prevented cartilage damage, as assessed using Kraus’ modified Mankin score and the Osteoarthritis Research Society International (OARSI) histopathology scoring system [97]. This was probably achieved through enhanced expression of lubricin, which is a mucinous glycoprotein, which coats the surface of cartilage to reduce friction [98]. The synovial lubricin level decreased in rats induced with OA early during the joint’s acute inflammation phase [97]. The rats receiving combination therapy showed an increase in synovial lubricin levels, concurrently with a reduction in interleukin-1 levels [97]. Individual treatments were less effective in preventing cartilage damage and increasing lubricin expression, indicating a synergistic effect between exercise and EVOO-enriched diets [97]. The content of EVOO used was not scrutinized in this study. 

Olive extract was also mixed with other compounds to explore their synergistic joint protective effects. Mével et al. supplemented olive and grape seed extracts in 10-week-old male C57/BL mice (4 g/kg/day) for four weeks, and 15-week-old female New Zealand white rabbits (100 mg/kg/day) for three weeks, before destabilizing their joints surgically (bilateral destabilization of the medial meniscus for mice; anterior cruciate ligament transection of the right joint for rabbits) [99]. The supplementation, which was standardized according to hydroxytyrosol (bioactive compound in olive) and procyanidins (bioactive compound in grape seed), continued for eight weeks for mice and ten weeks for rabbits [99]. Both mice and rabbits receiving treatment showed decreased OARSI scores and cartilage abrasion at the knee, relative to their negative controls [99]. In comparison, cartilage erosion to the mid-zone layer was observed in animals fed with glucosamine hydrochloride, indicating that olive and grape seed extracts showed better chondroprotective effects [99]. The decrease in aggrecan expression in the cartilage layer due to OA was prevented by the treatment [99].

Oleuropein, another major polyphenol in olives, was also investigated for its chondroprotective effects. Horcajada et al. compared the effects of a diet enriched with oleuropein (0.025% or 12.5 mg/kg body weight), rutin (a glycoside found in citrus fruit; 0.5% or 50 mg/kg body weight) or rutin and curcumin together (principal curcuminoid found in turmeric; 0.5%/0.25% or 50 mg/kg rutin and 125 mg/kg body weight curcumin) on the joints of four-week-old Dunkin–Hartley guinea pigs, for 31 weeks [100]. These animals developed OA spontaneously at 35-weeks of age [100]. The supplemented groups experienced varying degrees of improvement in global OA histological scores at the joints. Joint lesions of the groups supplemented with oleuropein and rutin at the femoral, tibial, medial and lateral compartments were less severe. Cellularity scores were lower in the oleuropein and rutin plus curcumin groups. Osteophyte scores were lower in the oleuropein group. Synovial scores were improved by oleuropein and rutin, and to a lesser degree, in the rutin plus curcumin supplemented group [100]. Changes in serum biochemical markers of OA are correlated with OA progression, whereby a higher level usually indicates elevated inflammation or cartilage degradation. In this study, all treatments could not suppress the serum nitrated collagen levels (cartilage degradation marker) due to OA. Oleuropein supplementation reduced the level of PGE_2_ (inflammation marker) and collagen-2 (cartilage degradation marker). Rutin alone, or in combination with curcumin, decreased collagen-2 and aggrecan levels (cartilage degradation marker) but did not affect PGE_2_ levels. Rutin, in combination with cucurmin, reduced fibulin-3 fragment levels (cartilage degradation marker) [100]. Overall, oleuropein and rutin individually showed potential anti-osteoarthritic effects, but synergistic effects were not seen in the rutin plus curcumin group.

In summary, olive extract, EVOO and polyphenols derived from olive trees possess potent chondroprotective effects, for example decreasing cartilage lesions and degradation in various animal models of OA. The underlying mechanisms could be due to decreased inflammation and enhanced lubricin expression. Cartilage regeneration by olive and its derivatives at the late stage of OA has not been studied so far. Late stage OA often requires surgical intervention, and physical rehabilitation and exercise have been recommended to help patients to regain function [101]. As shown by Musumeci et al., EVOO and physical activity act synergistically in preventing OA in rats [97]. Olive and its derivatives could be administered along with physical activity in rehabilitation programmes, to help OA patients regenerate cartilage and regain function [35]. This speculation should be validated in an animal study.

## 3. Effects of Olive and Its Derivatives in Human Studies

There have been four reports on the efficacy of olive and its derivatives on OA patients, of which three were randomized controlled trials [102,103,104] and one was a small-scale uncontrolled trial [105]. Subjects included in these studies were patients with OA [102,103,104,105]. An intervention, in the form of topical (olive extract and virgin olive oil) [103,105] or oral supplementation (olive extract and hydroxytyrosol) [102,104] was given. Comparison with a placebo [102,104] or an analgesic (piroxicam) [103] was performed in three of the clinical trials. Outcomes were measured using standardized OA questionnaires [102,103,104]. 

In a preliminary study, five subjects with symptomatic OA (aged 60.2 ± 8.1 years) applied 5 g of ointment, containing a 5% unsaponifiable fraction of unripe olive fruits, to their painful knee and hand joints, three times a day, for two–three weeks [105]. Weekly assessment of pain was performed using a visual analogue scale. Inflammation was inspected visually by physicians [105]. The unsaponifiable fraction contained lanosterol (2.60 mg/g oil), stigmasterol (2.15 mg/g oil), cycloartanol acetate (2.04 mg/g oil), stigmastan-3,5-diene (2.01 mg/g oil), obtusifoliol (1.93 mg/g oil), cholesta-4,6-dien-3-one (1.42 mg/g oil), α-amyrin (1.42 mg/g oil), α-tocopherol (1.32 mg/g oil), squalene (1.02 mg/g oil), β-amyrin (0.57 mg/g oil), and β-sitosterol (0.22 mg/g oil) [105]. The subjects experienced less joint pain and oedema, and improved mobility, one week after initiation of the treatment [105]. Redness and heat improved two weeks after the treatment. No adverse reactions were reported [105]. This pilot study provided some early evidence for the effects of olive derivatives on joint pain. However, it was a time-series study, without proper blinding, controls or randomization. The duration of treatment was relatively short (two–three weeks).

Following the aforementioned study, a double-blinded randomized clinical trial was performed on female Iranian osteoarthritic patients, aged 40–85 years [103]. The treatment group (*n* = 30) applied virgin olive oil topically, while the control group (*n* = 30) applied 1 g of piroxicam (NSAID) gel (0.5%) three times daily, for four weeks [103]. Both topical piroxicam and olive oil decreased Western Ontario and McMaster Universities Osteoarthritis Index (WOMAC) pain subscale scores and secondary outcome measures of the subjects [103]. The performance of olive oil was superior to piroxicam, starting at week two after initiation [103]. Only one patient complained of skin allergy after the olive oil application [103]. The dropout rate of this study was high, but the rate between treatment and controls was not significantly different. This might have affected the power of the study. In addition, the duration of treatment was relatively short [103]. 

Apart from topical application, the efficacy of olive extract supplement has also been studied. The effect of oral olive extract supplement in patients with OA (aged 55–75 years) was first tested by Bitler et al. in a randomized double-blinded placebo-controlled trial [102]. The treatment group (*n* = 30) took 400 mg of freeze-dried olive water extract orally for eight weeks. Osteoarthritic patients in the treatment arm showed significant improvements, as indicated by the Health Assessment Questionnaire-Disability Index, Disease Activity Score with 28-Joint Count Index [102]. 

Another randomized double-blinded placebo-controlled clinical trial tested the effects of oral hydroxytyrosol supplementation on knee OA. Subjects with knee pain were treated with 50.1 mg/day olive extract containing 10.04 mg hydroxytyrosol for four weeks (*n* = 13; aged 60.8 ± 7.2 years) [104]. They showed a higher improvement, based on Japanese Orthopaedic Association Scores, compared to the placebo group (total score) [104]. Scores for pain during sleeping at night were significantly reduced in the treatment group. Scores for pain during walking on a flat plane were improved marginally as well. Other types of pain were not improved significantly by hydroxytyrosol treatment [104]. In both oral supplementation studies, the number of participants was low and the duration of action was short [104]. 

A summary of the evidence from human clinical trials on the efficacy of olive and its derivatives is presented in Table 1. All the studies reviewed were small in sample size and short in duration. They used scoring systems to evaluate improvements in the subjects. More objective measurements, like joint space evaluation using X-ray images, could help to validate the efficacy of olive derivatives in improving OA. 

## 4. Molecular Effects of Olive and Its Derivatives in Cartilage Protection

Polyphenols from olive leaves and EVOO are postulated to exert their chondroprotective effects via anti-inflammatory actions. Nsir et al. pre-treated chondrocytes derived from OA patients and challenged with lipopolysaccharide (LPS, an inflammation inducer) with olive leaves and EVOO extracts of different polarities and maturities [106]. A chemical analysis showed that extract from olive leaves showed the highest degree of reducing power compared to various EVOOs [106]. All extracts did not induce cytotoxicity in the chondrocytes, and did not potentiate the effects of LPS [106]. Of note, EVOO derived from unripe olives displayed superior inflammatory activity, by abolishing the protein expression of iNOS, while the other extracts only reduced its expression [106]. Apolar fractions of EVOO could preserve the function of chondrocytes by maintaining their ability to produce collagen type-2, despite being challenged with LPS [106]. This may be due to the presence of lipid soluble substances, such as alpha-tocopherol and fatty acids, in the apolar fraction of EVOO. 

As an extension of their animal studies, Mével et al. treated rabbit chondrocytes with olive and grape seed extracts before exposing them to IL-1β [99]. The extract reduced the expression of iNOS, NO, cyclooxygenase-2 (COX-2) and PGE_2_ involved in inflammation, as well as cartilage degradation markers (MMP-13) [99]. The chondrocytes were also treated with sera of rabbits, fed with the extract at a dose of 500 mg/kg for eight days, to demonstrate that metabolites from the extract could be responsible for the observed chondroprotective effects [99]. The experiment showed that the sera prevented increases in NO, PGE_2_ and MMP13 due to interleukin-1 β stimulation [99].

Oleocanthal is an olive polyphenol which has gained considerable attention because it exerts effects similar to non-steroidal anti-inflammatory agents. Pre-treatment of ATDC-5 chondrocytes with oleocanthal and its derivatives protected chondrocytes from LPS-induced cell death at low concentrations (1–10 µM) [107]. This was achieved by reducing the expression of iNOS and subsequently the production of NO [107]. The actions of oleocanthal were accompanied by phosphorylation of p38 (which contributes either to cell survival or apoptosis) [107]. In contrast, this side effect was not seen with some oleocanthal derivatives [107]. The researchers indicated that the lack of p38 activation was beneficial, because it could cause apoptosis of other cells in the body. However, further study on additional pathways that govern the direction of p38 activation (survival/apoptosis) was not performed. The protein expression of inflammatory cytokines, such as IL-1β, TNF-α and granulocyte macrophage colony-stimulating factor in chondrocytes was also reduced by oleocanthal [108]. The effects of oleocanthal were also tested on macrophages since they are an important source of inflammatory cytokines [108]. Oleocanthal was found to reduce NO production by the murine macrophage, J774A.1, which is stimulated with LPS [108]. This was achieved by suppressing the protein expression of nitrite oxide synthase type-2 [108]. Oleocanthal also decreased the protein and gene expressions of macrophage inflammatory protein 1α and interleukin-6, in both macrophages and ATDC-5 chondrocytes [108]. 

Another olive polyphenol, hydroxytyrosol, is a potent antioxidant capable of modulating specific signalling pathways in chondrocytes. Previous reports showed that hydroxytyrosol reduced radical oxygen species production and associated DNA breakage induced by H_2_O_2_, in human primary chondrocytes [109]. It also prevented apoptosis of chondrocytes, induced by H_2_O_2_, by suppressing the increase in caspase-3 [109]. Increased inflammation (indicated by elevated COX-2 and iNOS), cartilage degradation (indicated by MMP-13), and terminal chondrocytic differentiation (indicated by runt-related transcription factor 2 (RUNX-2)) and vascular endothelial growth factor (VEGF) (induced by H_2_O_2_ and growth-related oncogene α (GROα)) were prevented by hydroxytyrosol [109]. mRNA expression of SIRT-1, an antiaging gene associated with OA, was lowered by GROα, but this was reversed with hydroxytyrosol treatment [109]. Later experiments showed that hydroxytyrosol increases the expression of SIRT-1 in primary human chondrocytes and C-28/I2 chondrocytes, with or without the presence of H_2_O_2_ [110]. This effect was modulated partially by microRNA-9 (miR-9), since pre-miR-9 transfection in chondrocytes partially reverses the beneficial effects of hydroxytyrosol in reducing cell death and caspase-3 when challenged with H_2_O_2_ [110]. The transfected cells also expressed higher levels of MMP-13, VEGF and RUNX-2, but their levels in cells treated with hydroxytyrosol remained low, compared to untreated cells [110]. 

Impaired autophagy has been associated with the pathogenesis of OA [111]. Autophagy removes impaired organelles and macromolecules in the chondrocytes to ensure they are healthy. Autophagy increases in chondrocytes during stress (early OA), but decreased autophagy is linked to cell death (late OA) [112]. Hydroxytyrosol could elevate protein expression of the autophagy marker, microtubule-associated protein 1A/1B-light chain 3 conjugate (LC3-II), and decrease the expression of p62 in chondrocytes, with or without the presence of H_2_O_2_ [113]. LC3-II is involved in the formation of the autophagy membrane, whereas p62 is a substrate used during autophagy [114]. Fluorescent staining also demonstrated an increase in late autophagy compartments (autolysosomes) in hydroxytyrosol-treated cells [113]. The addition of autophagy inhibitors completely abolished the protective effects of hydroxytyrosol on chondrocytes when challenged with H_2_O_2_ [113]. This effect was modulated by SIRT-1, because silencing of SIRT-1 abolished the antiapoptotic effects of hydroxytyrosol when chondrocytes were challenged with H_2_O_2_, and upregulated LC3-II expression [113]. However, silencing of SIRT-1 did not affect p62 expression [113]. Hydroxytyrosol influenced SIRT-1 by increasing its movement into the nucleus [113]. It was postulated that SIRT-1 in the nucleus catalysed the deacylation of LC3-II, which then crossed into the cytosol to initiate autophagy [113]. 

**Figure 1:**
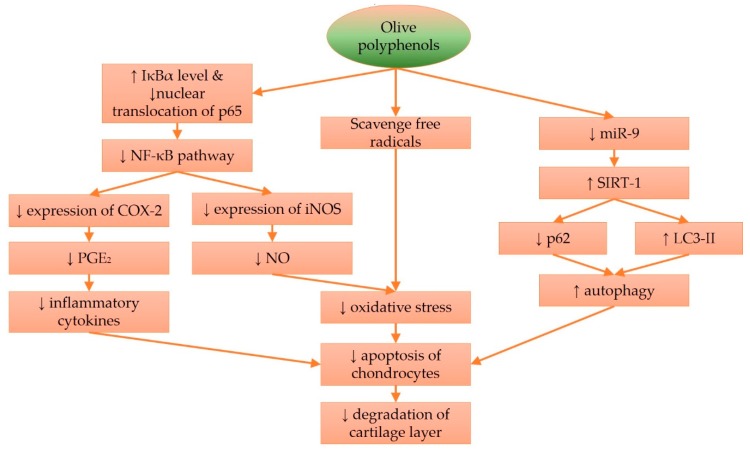
The mechanisms of action of olive polyphenols in preventing osteoarthritis. Abbreviations: COX-2 = cyclooxygenase-2; IκBα = nuclear factor of kappa light polypeptide gene enhancer in B-cells inhibitor, alpha; iNOS = inducible nitric oxide synthase; miR= microRNA; NFκB = nuclear factor kappa-light-chain-enhancer of activated B cells; LC3-II = microtubule-associated protein 1A/1B-light chain 3 conjugate, p62 = nucleoporin p62; p65 = transcription factor p65; PGE_2_ = prostaglandin E_2_; SIRT-1 = sirtuin-1.

In summary, olive oleocanthals possess anti-inflammatory activity by suppressing iNOS and NO production. Previous studies have shown that olive polyphenols, like hydroxytyrosol, can prevent degradation of the nuclear factor of kappa light polypeptide gene enhancer in B-cells inhibitor, alpha (IκBα) and prevent nuclear translocation of p65, thereby suppressing inflammation governed by the NFκB pathway [115,116]. Hydroxytyrosol also exerts more specific actions, such as reducing SIRT-1 expression and maintaining autophagy processes in chondrocytes and cell homeostasis, thereby preventing apoptosis of chondrocytes when challenged with oxidative or inflammatory assaults. These mechanisms prevent the breakdown of the cartilage layer and slow down the progression of OA (Figure 1). How far these in vitro processes mimic in vivo conditions, is debatable. Chondrocytes might behave differently when cultured in monolayer. Of all the cell culture studies summarized, only one grew the chondrocytes on a scaffold [109]. Only one study addressed the possibility that the metabolites, instead of the parent compounds derived from olive, exerted chondroprotective effects [99]. These are some of the issues to be resolved in the future. 

## 5. Perspectives on the Use of Olive and Its Derivatives in Combating OA

Pharmacokinetic studies have shown that polyphenols from virgin olive oil can be absorbed in realistic doses by humans [117]. The intestinal absorption of olive polyphenols, as represented by tyrosol and hydroxytyrosol levels, is around 55–56% in humans [118]. Oleuropein is metabolised and excreted as hydroxytyrosol [118]. The oral bioavailability of tyrosol and hydroxytyrosol is higher in olive oil compared to aqueous solutions [119]. Hydroxytyrosol undergoes extensive metabolism, mainly through glucoronidation processes in the liver [120]. Plasma concentrations of hydroxytyrosol peak at about 30 min and decrease henceforth [120]. The elimination half-life of hydroxytyrosol is 2.45 h [120]. The glucuronide conjugates of tyrosol and hydroxytyrosol are excreted in urine [121]. Olive polyphenols, or their metabolites, must be distributed to the respective body tissues to exert their biological effects. Since the cartilage layer is avascular, chondrocytes obtain nutrients from the synovial fluid. Data on the distribution of olive polyphenols in synovial fluid are limited. This is probably due to the invasiveness of the procedure involved, and the small volume of synovial fluid in animals.

Parkinson and Cicerale estimated that the average intake of olive polyphenols is 200 µg/day for humans practising traditional Mediterranean diets, assuming that 30–50 g of olive oil is consumed daily [122]. This is equivalent to a dose of 17.6 µg/kg body weight for rats or 10.3 µg/kg body weight for rabbits, based on the body surface ratio (km of a human with a body weight of 60 kg = 37; km of a large rat = 7; km of a rabbit = 12) [123]. In comparison, animal studies summarized in this review that demonstrated beneficial effects of olive polyphenols used much larger doses [94,99]. The study by Musumeci et al., which replaced the fat content of a standard diet with EVOO, was more similar to human consumption patterns. However, they failed to show beneficial effects in the group treated with EVOO alone [97]. This prompts the question whether the polyphenol content in normal olive oil consumption is sufficient to exert any chondroprotective effects, and whether supplementation at higher doses is required. 

Nevertheless, there are ways to improve the absorption of olive polyphenols using innovative delivery systems, through oral or topical routes. Ng et al. showed that fresh freeze-dried olive (containing around 3% hydroxytyrosol), delivered topically via an arginine bilayer film, could reduce arthritic scores, histological scores, paw and ankle circumferences and circulating interleukin-6 in a rat model of rheumatoid arthritis, induced by Freund’s adjuvant [124]. A topical formulation of hydrocortisone–hydroxytyrosol loaded chitosan nanoparticles has been developed to treat dermatitis [125]. Hydroxytyrosol loaded in an emulsion system could be delivered orally to enhance its bioavailability [126]. A gelled double emulsion system could reduce the degradation of hydroxytyrosol due to gastric acid and digestive enzymes [127]. 

Recent studies also highlight the role of adiponetins in the pathogenesis of OA [128,129,130]. Adipokines, including leptin, adiponectin and resistin, are expressed in adipose tissues, osteoclasts, osteoblasts and chondrocytes [128,131]. The exact role of these adipokines in OA is controversial. Leptin is higher in the synovial fluid of obese and OA patients [129,132]. Leptin was demonstrated to induce cartilage metabolism and inflammation via the induction of IL-1β, NO, PGE_2_, MMP-9 and MMP-13 expression [133,134]. However, leptin was also shown to increase chondrocyte proliferation, which contradicted its role as a proinflammatory molecule [129]. On the other hand, adiponectin levels are decreased in obese and diabetic patients [135]. This has been shown to downregulate IL-1β-induced MMP-13 expression and upregulate tissue inhibitors of MMP-2, to protect against OA [128,136]. However, adiponectin was also reported to induce the expression of iNOS and the release of IL-6, MMP-3 and MMP-9 from chondrocytes [128,137,138,139]. Diets based on olive oil were shown to increase adiponectin production by the adipocytes, and circulating adiponectin levels in overweight women. Thus, olive derivatives could influence the pathogenesis of OA via adiponectin, but this awaits validation [140,141]. 

This review is not without its limitations. Firstly, it only focuses on the anti-osteoarthritis effects of olive oil and its polyphenols. However, other compounds present in olive oil, such as alpha-tocopherol (the predominant form of vitamin E in nature), oleic and linoleic acids (both are the predominant unsaturated fatty acids in olive oil) also possess antioxidant and anti-inflammatory effects, which might contribute to the anti-osteoarthritic properties of olive oil [142,143]. All these compounds could act synergistically to provide better chondroprotective effects than olive polyphenols alone. The interaction of olive polyphenols with other food components, especially those found in Mediterranean diets, should also be studied, because of similar synergistic effects. Some studies summarised in this review have shown that procyanidins found in grape seeds might enhance the beneficial effects of olive polyphenols on cartilage [99]. Secondly, OA was involved not only in the degeneration of cartilage, but also in changes in subchondral bone, osteophyte formation, inflammation of synovium tissues and tendons, as well as muscle weakness [144]. Most of the mechanistic studies here focus on chondrocytes per se. Olive and its polyphenols are effective in preventing bone loss due to sex hormone deficiencies and chronic inflammation [145]. Therefore, they may also prevent destruction of subchondral bone during early OA [144]. Improving subchondral bone integrity has been proven to reduce the severity of OA [146]. There are limited human studies on the effects of olive and its derivatives on OA, and the available literature from the search has been included in this review. This highlighted the need for more well-planned human clinical trials to validate the role of olive and its derivatives in preventing OA. 

## 6. Conclusions

Olive and its derivatives show potential in preventing cartilage damage due to OA. This is attributed to their antioxidant and anti-inflammatory effects. In particular, hydroxytyrosol can modulate the SIRT-1 gene to improve autophagy and survival of chondrocytes. This review suggests that olive and its derivatives by themselves, or in combination with other approaches like physical activity, could be used to retard the progression of OA in individuals at risk. Current human studies suggest some improvements in the functional and pain scores in OA patients treated with olive extract, topically or orally, but more evidence from well-planned clinical trials to support the use of olive supplements in OA patients is required. Furthermore, the role of olive and its derivatives in late stage OA and post-surgical rehabilitation of OA patients require more research. 

## Figures and Tables

**Table 1:** A summary of the findings from human studies on the efficacy of olive and its derivatives against osteoarthritis.

Authors	Study Design	Patients, Interventions, Comparisons	Outcomes
Bitler et al. 2007 [102]	Randomized, double-blinded, placebo-controlled trial.	Patients with OA or RA, aged 55 to 75 years, free from other chronic diseases. Treatment group: 13 RAs and 30 OAs; 400 mg of freeze-dried olive water extract per day for 8 weeks Placebo group: 14 RAs and 33 OAs	OA patients receiving treatment showed significant improvements, as indicated by the Health Assessment Questionnaire–Disability Index, Disease Activity Score With 28-Joint Count index.
Bhoololi et al. 2012 [103]	Randomized, standard- controlled trial.	Female participants from a clinic in Iran, aged between 40–85 years diagnosed with OA. Treatment: 30 patients, virgin olive oil Control: 30 patients, 0.5% piroxicam 1 g gel, 3 times daily, for 4 weeks.	Both topical piroxicam and olive oil decreased WOMAC pain subscale scores and secondary outcome measures for the subjects. The performance of olive oil was superior compared to piroxicam, starting at week 2. Only one patient suffered a skin allergy after olive oil application.
Takeda et al. 2013 [104]	Double-blinded placebo- controlled trial.	Men and women with knee pain (gonarthrosis) Treatment group (13): aged 60.8 ± 7.2 years; 50.1 mg/day olive extract containing 10.04 mg hydroxytyrosol for 4 weeks. Placebo group (12): aged 61.4 ± 8.3 years.	Total improvement, based on JOA scores, was higher in the treated group compared to the placebo group, but not for subscales. Pain scores for pain during sleeping at night was significantly reduced for the treated group compared to the placebo group, pain during walking in flat planes was marginally signficiant, but other reductions in pain were not signficant.
Gelmini et al. 2015 [105]	Uncontrolled trial.	5 humans (men and women), 60.2 ± 8.1 years, diagnosed with symptomatic OA. They applied 5 g of oinment to their painful joints on knee and hands three times a day for 2–3 weeks. The oinment contained a 5% unsaponifiable fraction from unripe olive oil.	Joint pain, oedema and mobility started to improve after week 1. Redness and heat started to improve after week 2. No adverse reactions were reported.

Abbreviation: JOA = Japanese Osteoporosis Association; RA = rheumatoid arthritis; OA = osteoarthritis; WOMAC = Western Ontario and McMaster Universities Osteoarthritis Index.
